# MicroRNAs in ovarian follicular atresia and granulosa cell apoptosis

**DOI:** 10.1186/s12958-018-0450-y

**Published:** 2019-01-10

**Authors:** Jinbi Zhang, Yinxue Xu, Honglin Liu, Zengxiang Pan

**Affiliations:** College of Animal Science and Technology, Nanjing Agriculture University, Nanjing, 210095 People’s Republic of China

**Keywords:** MicroRNA, Follicular atresia, Granulosa cell apoptosis, Ovary

## Abstract

MicroRNAs (miRNAs) are short, noncoding RNAs that posttranscriptionally regulate gene expression. In the past decade, studies on miRNAs in ovaries have revealed the key roles of miRNAs in ovarian development and function. In this review, we first introduce the development of follicular atresia research and then summarize genome-wide studies on the ovarian miRNA profiles of different mammalian species. Differentially expressed miRNA profiles during atresia and other biological processes are herein compared. In addition, current knowledge on confirmed functional miRNAs during the follicular atresia process, which is mostly indicated by granulosa cell (GC) apoptosis, is presented. The main miRNA families and clusters, including the let-7 family, miR-23-27-24 cluster, miR-183-96-182 cluster and miR-17-92 cluster, and related pathways that are involved in follicular atresia are thoroughly summarized. A deep understanding of the roles of miRNA networks will not only help elucidate the mechanisms of GC apoptosis, follicular development, atresia and their disorders but also offer new diagnostic and treatment strategies for infertility and other ovarian dysfunctions.

**Electronic supplementary material:**

The online version of this article (10.1186/s12958-018-0450-y) contains supplementary material, which is available to authorized users.

## Background

Female fertility potential is based on the development and growth of ovarian follicles, and animal breeds with higher fecundity often show larger numbers of mature follicles in their ovaries and a higher ovulation rate than those with lower fecundity. However, the mammalian follicle utilization rate is extremely low because most follicles are removed from the ovaries before ovulation via a degenerative process known as atresia [1, 2]. Among domesticated animals, cows and sheep have approximately 1 million follicles, and sows have approximately 5 million primordial follicles in their ovaries at birth. However, more than 95% of these follicles undergo atresia [3–5]. Of the approximately 2 million primordial follicles that exist in human ovaries at birth, only up to 400 are ovulated throughout a female’s lifetime, which means that more than 99% of the follicles are lost. Therefore, methods to develop and utilize the abundant resources of ovarian follicles are consistently sought in animal reproduction research. Follicular atresia is a complex process that involves multiple regulatory factors, and studies on atresia mechanisms can be summarized in 3 aspects: 1) studies on follicular morphology and biochemistry, 2) studies on regulatory signalling pathways in granulosa cell (GC) apoptosis, and 3) studies on transcriptional/posttranscriptional mechanisms in GC apoptosis.

In 1987, Moor R et al. were the first to identify morphological changes in the atretic follicles of sheep [6], including the degree of translucency, vascularization of the follicle membrane and integrity of the membrane GC layers. Similar morphological features of isolated follicles were also observed in sows and cows [7, 8]. These features have been continually applied as important classification standards for atresia identification.

Endocrinological quantifications in follicular fluid are also standards for identifying antral follicle atresia. The concentration of oestrogen (E2) and the ratio between E2 and progesterone (P4) indicate a high correlation with the macroscopic classification of atresia. The steroid hormone concentrations detected by the enzyme immune assay (EIA) in medium-sized follicles (8–10 mm) in bovine follicular fluid suggest a P4/E2 < 1 in healthy follicles and a P4/E2 > 1 in atretic follicles. The ratio further drops with the degree of atresia [9]. A study on small (3–6 mm) and large (> 6 mm) ewe follicles using a nonextraction, solid-phase radioimmunoassay (RIA) suggested the same criteria [10]. In addition, steroid hormone concentrations detected in medium-sized (3–5 mm) pig follicles using RIA suggest a P4/E2 < 5 in healthy follicles and a P4/E2 > 5 in atretic follicles [11]. Based on our experience, a competitive ELISA method can also be used for hormone detection in pigs, and the classification standard is the same as that for RIA. Although the specific ratio varies depending on the species and detection method, all studies agree that higher E2 concentrations and lower P4/E2 levels indicate healthier follicular conditions.

With the development of ovarian studies, researchers have gradually realized that the basic physical mechanism of follicular atresia is GC apoptosis. When atresia occurs, pyknotic nuclei are first observed in GCs, which is followed by detachment of the GC layer and fragmentation of the basal membrane, ultimately resulting in hypertrophied thecal cells and disruption of thecal integration and thecal vessels. Degeneration of oocytes, however, may occur at any stage of atresia. The above phenomenon was first observed in bovine [12] and subsequently reported in chickens [13], rats [14], cows [15], sheep [16] and sows [17]. Therefore, follicular atresia studies have become increasingly focused on the molecular regulation of GC apoptosis. Further studies have shown that GC apoptosis may occur much earlier than the morphological changes in follicular atresia, which can be observed only when GC apoptosis reaches a certain degree [18, 19]. Generally, proliferation and differentiation of GCs leads to follicular maturation and ovulation, whereas apoptosis and degeneration of GCs results in follicular atresia [13, 20].

A considerable number of known molecular factors are involved in the regulation of GC apoptosis, including proapoptotic molecules, such as FAS (Fas cell surface death receptor), CASPs (caspases), MYC (MYC proto-oncogene BHLH transcription factor), TNF (tumour necrosis factor), F2RL3 (F2R-like thrombin or trypsin receptor 3, Par4) and PHB (prohibitin), as well as molecules that promote cell survival, such as activin, KITLG (KIT ligand, SCF), IFN (interferon), EDNs (endothelins), TP53 (tumour protein P53)b and NOBOX (newborn ovary homeobox-encoding gene) [21–24]. Although new regulatory factors are continuously being identified, comprehensive knowledge of the signalling networks that function during GC apoptosis remains limited.

In recent decades, the rapid rise of posttranscriptional mechanistic studies, especially miRNA studies, has brought a new perspective to reproductive system research. miRNAs comprise a broad class of endogenous, short, noncoding single-stranded RNA molecules that are normally 18–24 nt in length. Like mRNAs, miRNAs are originally transcribed from coding genes that are hundreds of kilobases long and occupy 1–3% of the genome [25]. miRNA-coding genes are distributed across chromosomes either individually or in clusters in which two or more miRNA genes are located within a short distance on the same segment of a chromosome. Thus, miRNA clusters on a given segment usually show a synergetic transcription pattern and related regulatory functions [26]. Based on gene structure, pri-miRNAs can be classified into three broad categories: Class I pri-miRNAs are transcribed independently of other genes, Class II pri-miRNAs are transcribed as an extension of a protein-coding gene, and Class III pri-miRNAs are transcribed as an extension of a noncoding RNA [27]. However, pri-miRNAs are poorly detected by standard methods, such as RT-PCR and RNA sequencing, due to rapid recognition and processing by the microprocessor complex, an enzyme arrangement made up of one Drosha protein and two DGCR8 proteins [28], resulting in 70–90 nt precursor miRNAs (pre-miRNAs) with hairpin structures. The pre-miRNAs are then transferred into the cytoplasm, and after further processing by Dicer, a cytosolic RNase III-type endonuclease, 18–24 nt double-stranded miRNAs are generated. With the elimination of one strand, the final single-stranded mature miRNA is loaded into an Argonaute (AGO) protein to form the miRNA-induced silencing complex (miRISC, RISC), which binds to specific sequences on target mRNAs, specifically to their 3′-untranslated regions (3′-UTRs), and results in either target mRNA degradation [29] or target mRNA transcriptional repression [30]. The estimation that nearly all mature sequences of coding mRNA transcripts contain miRNA response elements (MREs) implies crucial roles of miRNAs in almost all biological contexts during developmental, physiological and pathological processes. It is notable that one miRNA can regulate more than one gene, and conversely, a single gene can be regulated by multiple miRNAs [31].

In mammalian ovaries, folliculogenesis and atresia processes are tightly regulated by a complex network of genes. Therefore, miRNAs, which can originate from both cellular and extracellular sources, function as mediators of these processes via their extensive involvement in posttranscriptional mRNA regulation. The roles of ovarian miRNAs have been summarized in previous reviews on follicular and luteal development [32, 33], ovarian function [3, 34–36], and ovarian diseases and disorders [37–39]. Thus, this review primarily focuses on the latest findings concerning the roles of miRNAs in follicular atresia and GC apoptosis.

## Profile studies reveal specific shifts in miRNAs during atresia and other ovarian processes

Studies on ovarian miRNAs in humans, mice, cows, sows, sheep and goats have gradually assembled specific profiles of miRNA transcription in mammalian ovarian tissues. Ovarian miRNA profiles were also reported in studies on the reproduction of poultry animals, such as chicken [40] and geese [41]. High-throughput miRNA profiling studies have provided valuable references for deeply understanding the molecular mechanisms in ovarian tissue. Herein, we list the mammalian ovarian miRNA profiles that have been identified in whole ovaries, isolated follicles, or certain parts of follicles, including extracellular vesicles (EV) in follicular fluid, during various physiological and pathological processes (Table 1).

**Table 1:** miRNA profile studies carried out in mammalian ovaries

Tissue	Species	Description	Method	Reference
Ovary	bovine	foetal ovary	miRNA seq	[95]
	sheep	early/mid-gestational foetal ovary	PCR array	[96]
	mouse	newborn ovary	small RNA seq	[97]
	mouse	newborn ovary	microarray	[46]
	mouse	2-week-old/adult ovary	small RNA seq	[98]
	mouse	adult ovary	small RNA seq	[99]
	bovine	adult ovary	miRNA seq	[100]
	bovine	adult testicular and ovarian tissues of Holstein cattle	miRNA seq	[101]
	porcine	adult ovary and testis	miRNA seq	[102]
	sheep	ovaries of sheep under different nutritional status	small RNA seq	[103]
	human	adult ovary	small RNA seq	[104]
	mouse	ovaries of mice with/without cigarette smoke exposure	microarray	[105]
Follicle	bovine	during follicular development	microarray	[43]
	goat	multi/uniparous goat ovary	miRNA seq	[106]
	sheep	growing/preovulatory follicles/corpora lutea	miRNA seq	[45]
	porcine	healthy/early atretic/progressed atretic follicles	microarray	[42]
COC	bovine	COCs during late oogenesis	miRNA seq	[107]
Oocyte	human	oocytes during meiosis	microarray	[108]
GCs	human	cumulus GCs from women with and without PCOS	miRNA seq	[109]
	human	GCs from exogenous gonadotropins hyperresponders/normal responders	microarray	[110]
	human	GCs and serum from normal cycling and DOR women	microarray	[111]
	mouse	GCs from mice before and after an ovulatory dose of hCG treatment	microarray	[112]
	bovine	GCs of subordinate/dominant follicles	miRNA seq	[48]
	bovine	GCs of large/small follicles	microarray	[47]
Follicular fluid	human	PCOS patients and healthy controls	small RNA seq & PCR array	[113]
	human	PCOS patients and healthy controls	PCR array	[114]
	human	follicular fluid compares with plasma	PCR array	[115]
EV	bovine	EV from small/medium/large follicles	small RNA seq	[44]
	bovine	EV from GCs, COCs and their EVs	PCR array	[116]
Serum	human	serum from PCOS patients with IGM/NGT and healthy controls	PCR array	[117]
	human	serum from POF/normal women	microarray	[60]

Note: COC represents cumulus-oocyte complexes, EV represents extracellular vesicles

**Fig. 1:**
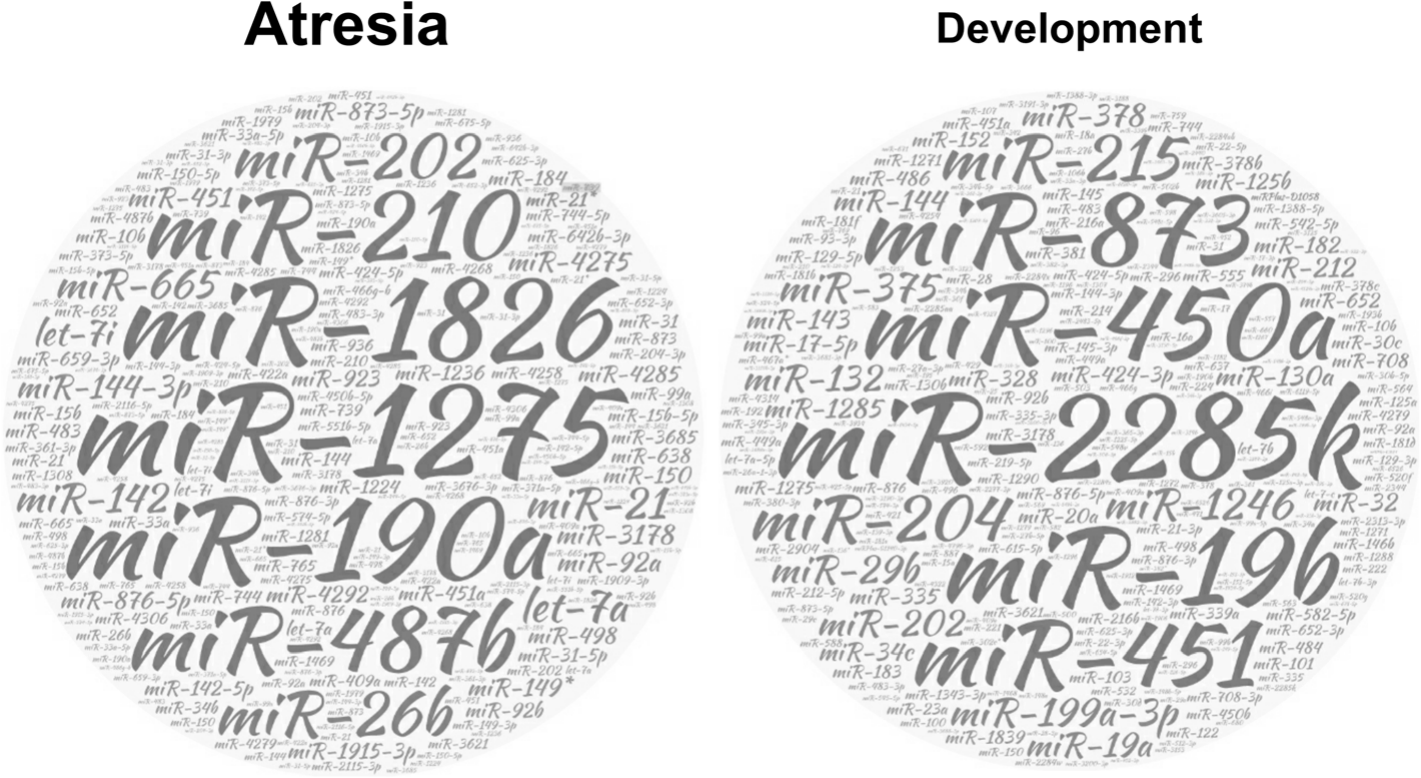
A glance at the main miRNAs that play roles in atresia and follicular development processes. Note: a larger font size represents a higher frequency of a certain miRNA that was reported in related studies

These studies applied microRNA sequencing, microarray or PCR array methods and provided useful candidates for further studies, which may lead to the identification of biomarkers for medical diagnosis in ovarian disorders. To gain a general view of miRNA functions in ovaries, we generated 2 cloud charts (Fig. 1) based on previously performed high-throughput studies by simply integrating the results of research on 1) miRNAs differentially expressed during follicular atresia [42, 43] and 2) miRNAs differentially expressed among small, medium-sized and large follicles [43–48]. Clearly, miRNAs that are involved in different biological processes are remarkably divergent in the ovary. Among the most notable miRNAs that changed during atresia, miR-1275, which is known to regulate insulin-like growth factor-2 mRNA-binding proteins (IGF2BP1, IGF2BP2 and IGF2BP3) in cancer [49], was recently shown to affect E2 synthesis and lead to GC apoptosis by targeting liver receptor homologue (LRH)-1 [50]. In addition, although not yet related to ovarian atresia, miR-1826 was reported to regulate β-catenin (CTNNB1) by the observance of direct binding in cancer studies [51], while miR-190a was shown to mediate VEGF [52] and oestrogen receptor (ER)-related signalling in angiogenesis [53], and miR-210 was proven to be related to hypoxia [54, 55]. It is clear that high-frequency miRNAs are connected to atresia-related pathways and can thus be considered helpful hits in follicular atresia and cell apoptosis-related studies.

## Specific miRNAs and their functions in follicular atresia and GC apoptosis

To explore the effective functions of miRNAs during atresia, gain- and loss-of-function studies have been applied by researchers in vitro. A list summarizing known studies on miRNAs directly or indirectly involved in GC proliferation and apoptosis is presented in Table 2, which includes combinations of miRNAs, their verified target genes and miRNA functions. The list will certainly continue to grow with contributions from research on this topic. Here, we attempt to summarize several main families and groups of miRNAs that play critical roles in GC apoptosis and the follicular atresia process.

**Table 2:** miRNAs, target genes and their functions in GC apoptosis and follicular atresia

miRNA	Species	Target	Function	Reference
let-7 g	porcine	*MAP3K1*	promotes GC apoptosis	[61]
let-7 g	porcine	*TGFBR1*	promotes GC apoptosis	[58]
miR-10b	goat	*BDNF*	suppresses GC proliferation	[118]
miR-15a	human	unknown	promotes release of progesterone and testosterone	[91]
miR-21	mouse	unknown	inhibits apoptosis, increases ovulation rate	[119]
miR-22	mouse	*Sirt1*	inhibits GC apoptosis	[120]
miR-23a	human	*SMAD5*	promotes GC apoptosis	[78, 121]
miR-26b	porcine	*ATM*	promotes GC apoptosis	[42]
miR-26b	porcine	*SMAD4*	promotes GC apoptosis	[64]
miR-26b	porcine	*HAS2*	promotes GC apoptosis	[122]
miR-27a	human	*SMAD5*	promotes GC apoptosis	[78]
miR-34a	porcine	*INHBB*	promotes GC apoptosis	[123]
miR-34c	porcine	unknown	proapoptotic and antiproliferative factor	[124]
miR-92a	porcine	*SMAD7*	inhibits GC apoptosis	[65]
miR-93	human	*CDKN1A*	promotes GC proliferation	[125]
miR-106a	human	*apoptosis signal-regulating kinase 1* (*ASK1*)	reduces GC viability and promotes apoptosis	[111]
miR-125a-5p	mouse	*Stat3*	promotes GC apoptosis	[126]
miR-126*	porcine	*FSHR*	promotes AR-induced GC apoptosis	[127]
miR-146a	human	*IRAK1/TRAF6*	promotes GC apoptosis	[128]
miR-181b	porcine	*SMAD7*	inhibits GC apoptosis	[67]
miR-182	rat	*Smad7*	inhibits GC apoptosis	[66]
miR-224	mouse	*Smad4*	inhibits GC proliferation and E2 release	[63]
miR-320	mouse	*E2f1/Sf-1*	inhibits E2 synthesis and GC proliferation	[129]
miR-378-3p	bovine	*PGR*	inhibits GC differentiation	[130]
miR-378	porcine	*CYP19A1*	decreases E2 production	[88]
miR-383	mouse	*Rbms1*	enhances E2 release from GCs	[131]
miR-503/322/351 Cluster	mouse	Autophagy/Mitophagy-Associated Genes	reduces of mitochondrial activity in GC	[132]
miR-764-3p	mouse	*Sf-1*	decreases steroidogenesis	[89]
miR-1275	porcine	LRH-1	represses E2 synthesis	[50]

### Let-7 family in follicular atresia

The let-7 family is one of the first discovered miRNA groups, and its family members are highly conserved in sequences across animal species [56]. The functions of let-7 family members include cell proliferation, differentiation, tissue development and tumour suppression [57]. Both microarray and RT-qPCR analyses have shown that members of the let-7 family are differentially expressed during porcine follicular atresia. For example, the expression levels of let-7a, let-7b, let-7c, and let-7i were decreased in early and progressed atretic follicles compared to those in healthy follicles [58, 59]. Downregulation of let-7c was also detected in premature ovarian failure (POF) patients compared to that in normal women [60], which implied that let-7c plays a positive role in healthy follicular development. The function of let7-g in follicles appears to be different from that of other family members because it is highly expressed during atresia [59]. Further studies have identified the anti-apoptotic genes *MAP3K1* (mitogen-activated protein kinase kinase kinase 1) and *TGFBR1* (transforming growth factor-β type 1 receptor) as direct targets of let-7 g. The let-7 g-mediated suppression of MAP3K1 results in the expression and dephosphorylation of the transcription factor *FoxO1* (forkhead box O1), which accumulates in the nucleus and induces GC apoptosis [61]. Furthermore, a let-7 g-induced blockage of *TGFBR1* increases caspase-3 activity and the apoptosis rate due to suppression of the *TGFβ* signalling pathway. In addition, let-7 g may function as a potent antagonist that regulates the same pathways as let-7a/b/c/I, which makes the let-7 miRNA family a bidirectional regulatory network during GC apoptosis [58]. In brief, the let-7 miRNA family shows great potential in the regulation of follicular atresia; the detailed mechanisms await further validation.

### TGFβ/SMAD signalling and the miR-17-92 cluster in follicular atresia

The TGFβ signalling pathway has a wide spectrum of functions that depend on specific biological contexts. Generally, TGFβ ligands (TGFB1, TGFB2, and TGFB3) first activate a membrane receptor serine/threonine kinase complex composed of type II (TGFBR2) and type I (TGFBR1) receptors. After phosphorylation by TGFBR1, SMAD2/3 forms an oligomeric complex with SMAD4 and translocates to the nucleus, where it can either promote or inhibit the transcription of target genes [62]. In follicular studies, the roles of the TGFβ pathway and related miRNA regulation have been frequently reported in recent years. It is known that miR-224 and miR-26b regulate the pathway by targeting *SMAD*4 [63, 64]; miR-23a and miR-27a promote human GC apoptosis by targeting *SMAD5*, as described above, while miR-92a, miR-181b and miR-182 directly bind to *SMAD7* [65–67], which is considered an antagonist of the TGFβ pathway [68] and an amplifier of TGFβ-induced apoptosis [69]. Interestingly, miR-92a belongs to the miR-17-92 cluster, which includes miR-17, miR-18a, miR-19a, miR-19b, miR-20a, and miR-92a and is activated via directly binding the *MYCN/MYC* promoter. Although other members of the cluster have rarely been reported in the ovary, the cluster has been shown to regulate the TGFβ pathway and affect apoptosis in many biological processes, including tumourigenesis and normal development of the heart, lungs, and immune system [70]. Studies on cancer cell lines suggest that the cluster acts as a potent inhibitor of the TGFβ pathway [71] by directly binding to multiple pathway components, especially SMADs in neuroblastoma [72]. The cluster may also mediate the antiproliferative effect of histone deacetylase inhibitors via the proapoptotic protein BIM (Bcl-2-interacting mediator of cell death) [73]. Further, studies on miRNA-mediated TGFβ/SMAD signalling may provide a comprehensive understanding of the TGFβ/SMAD signalling regulation and functions in GC apoptosis and follicular atresia.

### miR-23-27-24 cluster, miR-183-96-182 cluster and other potential miRNA clusters

Close evolutionary, transcriptional and functional relationships among related homologous/clustered miRNAs have been revealed by bioinformatics analyses [74]. The miR-23-27-24 cluster comprises the miR-23a gene cluster (including the miR-23a, miR-27a and miR-24-2 genes) and the miR-23b cluster (including the mir-23b, mir-27b and mir-24-1 genes), which are located on chromosomes 19(−) and 9(+), respectively, in the human genome. Studies have suggested that the miR-23-27-24 cluster plays roles in various biological and pathological processes, including erythropoiesis [75], angiogenesis [76], cell invasion and hepatic metastasis [77]. In human ovarian follicles, upregulation of mir-23a and mir-27a was observed in POF patients [60]. A functional study showed that *SMAD5* is a direct target of both mir-23a and mir-27a, which promote GC apoptosis via the Fas-FasL pathway [78]. These observations suggest that miRNA clusters play a role in follicular atresia. Clearly, the functions of other members of the miR-23-27-24 cluster and whether they share coordinate function in the process of follicular atresia deserve further research.

The miR-183-96-182 cluster is highly conserved, and its members are located within a 5 kb region on human chromosome 7q32.2 [79]. Expression of the miR-183-96-182 cluster has been reported in both follicular and luteal cells. The expression levels of members of this cluster are higher in preovulatory dominant follicles than in subordinate follicles [48] and further increased in luteal tissues [80]. Members of the miR-183-96-182 cluster were validated to target the 3’-UTR of the FOXO1 gene, an important transcription factor for follicle-stimulating hormone responsive genes in ovarian GC [81, 82], and thus regulate follicular and luteal development via exerting effects on cell survival and steroid production. miR-182 was also reported to inhibit GC apoptosis by targeting *SMAD7* [66]. It has been reported that the miR-183-96-182 cluster is regulated by the Wnt/β-Catenin pathway via direct interaction between *CTNNB1* and the promoter region of its coding gene. In addition, the miR-99b/let-7e/miR-125a cluster, which works co-ordinately to regulate ARID3A (AT-rich interaction domain 3A) in oesophageal squamous cell carcinoma [83], and the miR-106a-363 cluster (miR-20b, miR-106a, miR-363-3p, and miR-363-5p), which inhibits the proliferation of oral squamous carcinoma cells by decreasing the expression of several sibling miRNAs encoded by the miR-17-92 or miR-106b-25 cluster [84], may also play roles in follicular atresia, as other members of the let7 family and miR-106a alone have been shown to function in the atresia process.

### miRNAs and steroidogenesis

Gonadal hormones, such as oestrogen and progesterone, play significant roles in both the endocrine and intracrine regulation of all aspects of female reproduction. In ovaries, the miRNA regulation of steroidogenesis and hormone secretion has a direct impact on the follicular atresia process. The steroidogenic acute regulatory (STAR) protein facilitates cholesterol transport to supply substrates for steroid hormone biosynthesis. Although let-7 [85] and miR-150 [86] were reported to directly inhibit *Star* at the post-transcriptional level in Leydig cells, the only miRNA known to regulate *Star* in ovary is miR-133b, which directly targeting *Foxl2* thus inhibits the *Foxl2*-mediated transcriptional repression of *Star* and *Cyp19a1* to promote oestradiol production [87]. When it comes to the main aromatase CYP19A1, miR-378 reduces oestrogen synthesis by directly binding to *CYP19A1* [88]. Other miRNAs that indirectly regulate CYP19A1 include miR-320 and miR-383, which directly target *E2F1/SF1* and eventually affect the expression of their downstream gene *CYP19A1*; miR-764-3p, which directly binds to *SF1* [89]; and miR-1275, which targets another *CYP19A1* transcription factor, LRH1 [50]. In a spermatogenesis study, the long noncoding RNA NLC1-C was reportedly associated with testicular embryonal carcinoma cell proliferation by functioning as a miR-320a/383-sponge that repressed miR-320a and miR-383 transcripts [90]. This mechanism may be worthy of further exploration in the ovary. However, the miRNAs involved in progesterone synthesis have not been studied as extensively. miR-15a was reported to promote the release of progesterone and testosterone, but the target gene and regulatory mechanism are unclear [91]. The relationship between alterations in steroidogenesis and GC apoptosis has been demonstrated in several studies [92]. Our transcriptomic study on healthy and early atretic follicles also suggested a major role of steroidogenesis in atresia initiation [93]. Therefore, a deeper understanding of miRNA-mediated steroidogenesis regulation will be a promising area in follicular development and atresia research.

### Bioinformatic predictions of key miRNA-mediated pathways in the atresia process

To further explore possible miRNA-mediated pathways involved in follicular atresia, we applied bioinformatics integration and mining of existing functional miRNAs. All known atresia-related miRNAs (from Table 2) were used as starting points. Their target genes, which were experimentally confirmed by a luciferase reporter assay, were screened using miRTarBase (http://mirtarbase.mbc.nctu.edu.tw/php/search.php) [94], and 426 genes were obtained (Additional file 1: Table S1). Then, we performed functional enrichment analyses of target genes using the Cytoscape plug-in ClueGO and the latest KEGG, REACTOME and WikiPathways databases (Additional file 2: Table S2, Additional file 3: Table S3, Additional file 4: Table S4). The functional enrichment generated from KEGG showed that most highlighted pathways were related to cancers of various types, largely because miRNA studies have mostly focused on cancer models. Nonetheless, excluding cancer-related pathways, signalling pathways that include TGF-β, PI3K-Akt, p53, FoxO, TNF, Toll-like receptor, HIF-1, WNT VEGF, oestrogen, progesterone and insulin mediation appear to play roles in the processes of follicular atresia and GC apoptosis. These results mostly coincide with known signalling pathways that function in ovarian development, maturation and atresia. Detailed information can be found in the supplementary tables.

## Conclusions

miRNA-mediated regulation is now widely valued in ovarian function studies. Although the mechanisms of follicular atresia have not been precisely defined, recent reports have demonstrated a causal link between the levels of certain miRNAs and the fate of GCs/follicles in several species. Profiling studies have demonstrated that diversified miRNAs play roles in various ovarian physiological and pathological processes. In the specific area of follicular atresia and GC apoptosis, some miRNAs are known to work individually to regulate particular genes, while others function in clusters to finely adjust certain signalling pathways or cellular processes. However, in both cases, the function of miRNAs should not be viewed as a single factor but as part of a regulatory network that involves hormones, growth factors and other regulatory noncoding RNAs from each component of the follicle. The ultimate functions of miRNAs on certain target genes can be affected by multilevel regulatory networks, from the transcriptional regulation of their own coding genes to interference from other competing endogenous RNAs, to achieve timely adjustment in cells. Therefore, the following areas may attract more attention in future studies: 1) competition and cooperation of regulatory noncoding RNAs, such as miRNAs, lncRNAs (long noncoding RNAs) and piRNAs (Piwi-interacting RNAs); 2) communication among miRNAs generated from different parts of the follicle, including oocytes, GCs (from the cumulus or near basement membrane) and thecal cells; 3) influence of single-nucleotide polymorphisms (SNPs) on miRNA genes that are involved in ovarian pathological processes; and 4) functions of small RNAs in EV. In addition, with the development of high-throughput methods, comprehensive studies on noncoding RNA networks and interactions with their targets will lead to a deeper understanding of miRNA functions in ovarian follicular atresia. The advancement of our knowledge of miRNA-mediated networks will not only help elucidate the mechanisms of GC apoptosis, follicular development, atresia and other disorders but also provide potential uses for future clinical diagnosis and treatment.

## Additional files

Additional file 1: **Table S1.** (XLSX 13.9 kb)

Additional file 2: **Table S2.** (XLS 85 kb)

Additional file 3: **Table S3.** (XLS 198 kb)

Additional file 4: **Table S4.** (XLS 82 kb)

## Abbreviations

COC: Cumulus-oocyte complexes
EV: Extracellular vesicles
GC: Granulosa cell
lncRNAs: Long noncoding RNAs
miRNAs: MicroRNAs
PCOS: Polycystic ovary syndrome
piRNAs: Piwi-interacting RNAs
POF: Premature ovarian failure
